# Metal–Organic-Framework-Derived Ball-Flower-like Porous Co_3_O_4_/Fe_2_O_3_ Heterostructure with Enhanced Visible-Light-Driven Photocatalytic Activity

**DOI:** 10.3390/nano12060904

**Published:** 2022-03-09

**Authors:** Qi Cao, Qingqing Li, Zhichao Pi, Jing Zhang, Li-Wei Sun, Junzhou Xu, Yunyi Cao, Junye Cheng, Ye Bian

**Affiliations:** 1 Key Laboratory of Energy Thermal Conversion and Control of Ministry of Education, School of Energy and Environment, Wuxi Engineering Research Center of Taihu Lake Water Environment, Southeast University, Nanjing 210096, China; 213151155@seu.edu.cn (J.Z.); liwei-sun@seu.edu.cn (L.-W.S.); 213193744@seu.edu.cn (J.X.); 2 Department of Chemistry, College of Sciences, Nanjing Agricultural University, Nanjing 210095, China; 2019203072@njau.edu.cn; 3 State-Operated Wuhu Machinery Plant, Wuhu 241099, China; pzc0279@163.com; 4 Department of Intelligent Development Platform, Laundry Appliances Business Division of Midea Group, Wuxi 214028, China; caoyy12@midea.com; 5 School of Science and Engineering, The Chinese University of Hong Kong, Shenzhen 518172, China

**Keywords:** MOF derivative, cobalt oxide, iron oxide, hierarchical heterostructure, photocatalysis

## Abstract

A porous ball-flower-like Co_3_O_4_/Fe_2_O_3_ heterostructural photocatalyst was synthesized via a facile metal–organic-framework-templated method, and showed an excellent degradation performance in the model molecule rhodamine B under visible light irradiation. This enhanced photocatalytic activity can be attributed to abundant photo-generated holes and hydroxyl radicals, and the combined effects involving a porous structure, strong visible-light absorption, and improved interfacial charge separation. It is notable that the ecotoxicity of the treated reaction solution was also evaluated, confirming that an as-synthesized Co_3_O_4_/Fe_2_O_3_ catalyst could afford the sunlight-driven long-term recyclable degradation of dye-contaminated wastewater into non-toxic and colorless wastewater.

## 1. Introduction

Various pollutants in water environments can directly cause serious harm to the lives and health of human beings, animals and plants. Organic dyes, for example rhodamine B (RhB), methylene blue and methyl orange, as one of the most common industrial pollution sources at present, have attracted tremendous attention because of their geno- and ecotoxicity [1,2,3,4,5,6]. Therefore, the development of water treatment technologies regarding dye degradation has become a top priority. Among various methods, photocatalysis is recognized as one green and efficient alternative for organic pollutant degradation, where its key issue lies in the facile preparation of highly active and stable photocatalysts [7,8,9,10].

As one of the most promising multi-functional materials, metal–organic frameworks (MOFs) are often considered to be novel photocatalysts due to their abundant and editable active sites and large surface area. However, some of their defects, such as poor light absorption and metal ion leaching due to an unstable structure, may seriously limit their practical applications [11,12,13]. In order to solve these problems, in this study, a flower-like cobalt 2,5-thiophenedicarboxylic coordination polymer (Co-TDC) was used as a template to synthesize a novel Co_3_O_4_/Fe_2_O_3_ heterostructural photocatalyst with improved light harvesting and photocatalytic performance. The facile preparation, structural versatility, and superior dye degradation performance of this Co_3_O_4_/Fe_2_O_3_ heterostructure provides new inspirations for the development of higher-performance photocatalysts towards water environment remediation.

## 2. Materials and Methods

### 2.1. Synthesis of Ball-Flower-like Porous Co_3_O_4_/Fe_2_O_3_ Heterostructure

Briefly, 0.1 g of Co-TDC (Sinopharm Group Co., Ltd., Shanghai, China) and 0.0482 g of FeCl_3_·6H_2_O (Sinopharm Group Co., Ltd., Shanghai, China) were added into 5 mL of deionized water. After 30 min of ultrasonic treatment, the mixture was dried at 60 °C for 12 h. Afterwards, the obtained powder was calcined at 550 °C for 2 h. The final Co_3_O_4_/Fe_2_O_3_ product is denoted as CF in this work for convenience.

### 2.2. Characterization

The chemical composition and phase structure of samples were analyzed by X-ray powder diffraction (XRD, SmartLab^®^ by Rigaku, Tokyo, Japan). The morphology was recorded using field-emission scanning electron microscopy (SEM, JSM-7800F by JEOL, Japan) and transmission electron microscopy (TEM, JEM-2100F by JEOL, Japan). X-ray photoelectron spectroscopy (XPS, EscaLab 250Xi by Thermo Fisher Scientific, Waltham, MA, USA) was performed to investigate element distribution and valence states. The magnetism and optical properties of samples were studied using vibrating sample magnetometer (VSM, LakeShore7404 by Quantum Design, San Diego, CA, USA) and diffuse-reflection spectroscopy (DRS, Cary-5000 by Agilent, Santa Clara, CA, USA).

### 2.3. Photocatalysis Measurements

The adsorption and photocatalysis processes of as-prepared catalysts were evaluated by the degradation of RhB in an aqueous solution under visible light irradiation at room temperature (ca. 25 °C). A 500 W xenon lamp with a cut-off filter (*λ* > 420 nm) was used to generate visible light. Amounts of 0.1 g of catalyst powder and 50 mL RhB aqueous solution (initial solution pH ≈ 4) were added to a 100 mL quartz tube and continuously stirred during the degradation experiment. Before irradiation, the reaction solution was magnetically stirred in the dark for 30 min to reach complete adsorption/desorption equilibrium. During the photocatalytic experiment, 5 mL reaction solution was extracted every 10 min, and the concentration of residual RhB was determined by measuring its absorbance at 590 nm on a UV-visible spectrometer (UV-3600i Plus by Shimadzu, Kyoto, Japan). The 5 mL solution was added back into the reaction solution after measurement.

## 3. Results and Discussion

**Figure 1:**
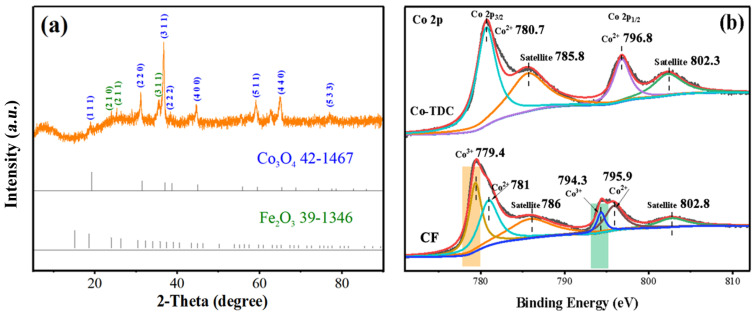
XRD pattern (**a**) and Co 2p XPS spectra (**b**) of as-synthesized CF catalyst.

The chemical composition and crystal structure of CF were analyzed by XRD. As shown in Figure 1a, the characteristic diffraction peaks located at 19.1°, 31.2°, 36.8°, 44.7°, 59.1°, and 65.1° could be attributed to Co_3_O_4_ (PDF#42-1467), while the other peaks at 35.6° and 62.9° could be assigned to Fe_2_O_3_ (PDF#39-1346), indicating that the as-prepared sample was composed of Co_3_O_4_ and Fe_2_O_3_. The chemical states of the sample surface were further analyzed by XPS. Considering the sample preparation method, only cobalt element was studied emphatically. In Co 2p spectra (Figure 1b), the asymmetric peaks at around 780.7 eV and 796.8 eV, and shake-up type satellite peaks at 785.8 eV and 802.3 eV of Co-TDC, could be well-indexed to Co^2+^, implying that cobalt in Co-TDC was only in the form of Co (II). On the other hand, for CF, two new peaks could be identified at around 779.4 eV and 794.3 eV, which were both ascribed to Co^3+^ [14,15,16,17,18]. This revealed that Co^2+^ in Co-TDC was partially oxidized to Co^3+^ during calcination, and thus Co_3_O_4_ was obtained as a result. Meanwhile, Fe 2p spectrum of CF was also recorded, as shown in Figure S1. It was revealed that Fe^3+^ ions were still dominant, which corresponded to the Fe_2_O_3_ phase. However, the minor peak at around 732.2 eV suggests that a little Fe^3+^ was reduced to Fe^2+^ along with the oxidation of Co^2+^ to Co^3+^ [19,20,21,22,23].

**Figure 2:**
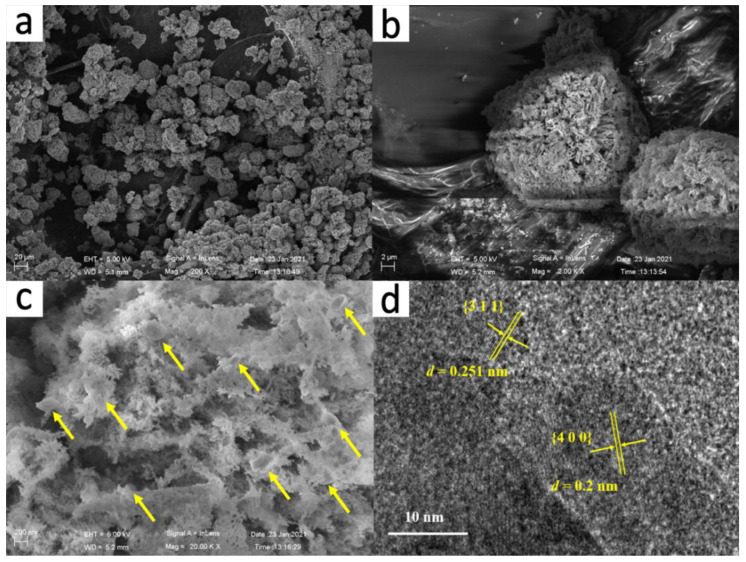
Representative SEM images (**a**–**c**), and HRTEM image (**d**) of the CF catalyst.

SEM images of the CF heterostructure are shown in Figure 2a–c. It can be observed from Figure 2a,b that CF has a regular ball-flower-like morphology with a spherical size ranged at 10–20 μm, which was retained from the Co-TDC template, as shown in Figure S2 of the Supplementary Materials. It is worth noting that the sheet-like fundamental units of Co_3_O_4_ in CF became much more porous after calcination, with large numbers of ~200 nm Fe_2_O_3_ nanoparticles (Figure S3) embedded within the pores, as indicated by the yellow arrows in Figure 2c, which facilitate the adsorption and degradation of dye molecules on the surface. Moreover, the elemental mapping profiles in Figure S4 also help to verify that the distribution of Fe_2_O_3_ within highly porous Co_3_O_4_ is uniform while it is random. In order to further determine the chemical composition of the synthesized catalyst, HRTEM image was also recorded, as shown in Figure 2d. The identified two lattice fringes with an interval of 0.25 and 0.20 nm could be indexed to the (311) facet of Fe_2_O_3_ and (400) facet of Co_3_O_4_, respectively, which is in good agreement with the XRD result.

**Figure 3:**
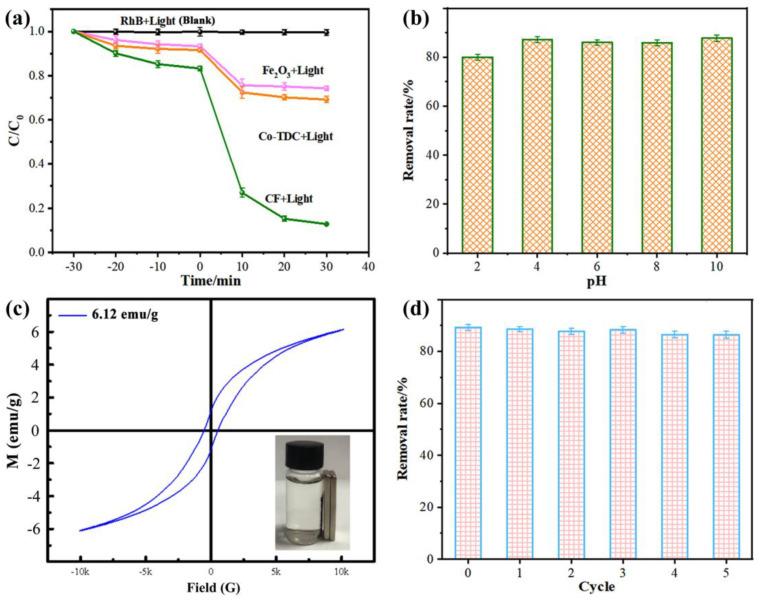
(**a**) RhB degradation efficiencies of different samples; (**b**) the effect of pH of reaction solution; (**c**) VSM curve of CF; (**d**) recyclability of CF photocatalyst for RhB degradation.

The degradation efficiencies of different samples for RhB are displayed in Figure 3a. When the catalyst was not present in the solution, RhB could hardly undergo self-degradation under visible light (i.e., black plots). The reaction solution was first stirred in the dark for 30 min for the catalyst–RhB interface to reach the adsorption/desorption equilibrium. Typically, the contribution of RhB removal by adsorption is lower than 20%, which is in proportion to the surface area of the catalyst. In photocatalysis systems, CF demonstrated a superior performance than Co-TDC and Fe_2_O_3_, indicating that CF possesses the highest photocatalytic activity. This could be explained by the following aspects: (i) The highly porous structure of CF provided abundant active sites, as revealed in Figure 2b,c [24,25,26]; (ii) The *p*-*n* heterojunction that formed between Fe_2_O_3_ and Co_3_O_4_ could promote the separation of photo-generated electron and hole pairs [27,28,29,30]. The promoted charge separation, and thus the inhibited charge recombination, was witnessed by the significantly decreased photoluminescence (PL) intensity of CF composites compared to pristine Fe_2_O_3_ particles, as displayed in Figure S5 of the Supplementary Materials [31,32,33,34,35,36,37,38]. The variation in the RhB degradation efficiency of CF in different pH conditions is presented in Figure 3b, suggesting that the catalyst could maintain a superior photocatalytic degradation activity in the pH range of 4–10, despite the fact that the degradation rate decreased to a certain extent in a strong acid environment (pH ≤ 2). This may be due to the dissolving of oxides by strong acid, resulting in a loss of active material in the CF catalyst for the degradation of RhB. However, considering that the actual surface water or groundwater is mostly weakly acidic or weakly alkaline, the CF catalyst is still applicable to the oxidative degradation of organic pollutants in natural water bodies.

The service life of a catalyst is an important technical indicator for evaluating its potential for practical usage. After the reaction, the catalyst in the solution could be easily and quickly separated due to its magnetism, as revealed in Figure 3c. Then, the recycled CF catalyst was rinsed with ethanol solution to remove the residual organics on the surface. Afterwards, it could be reused for RhB removal under the same conditions, as presented in Figure 3d. An excellent degradation efficiency of >86% was achieved after the CF catalyst was recycled and reused for five cycles, which maintained about 97% efficiency of the initial cycle (i.e., ~89.1%), confirming the recyclability of CF for long-term dye degradation in practical wastewater treatment.

**Figure 4:**
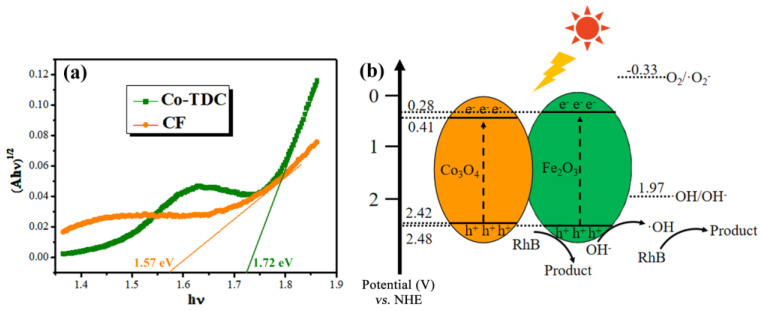
(**a**) Tauc plots, i.e., plots of (*αhν*)^0.5^ vs. photon energy (*hν*), derived from diffuse-reflectance spectra of the Co-TDC and CF samples; (**b**) Band alignment and photocatalytic mechanism of the CF heterojunction under visible light illumination.

Figure 4a displays the optical absorption of samples. It is observed that the absorption of Co-TDC is far lower than CF in the visible-light band. The CF catalyst maintains a superior absorption in the range of 550–750 nm, suggesting its capability for a visible-light-driven photocatalytic reaction. In addition, the threshold wavelengths of Co-TDC and CF are determined to be 619 nm and 685 nm, respectively. The corresponding bandgap and conduction band (*CB*)/valence band (*VB*) position can be calculated according to the following formulas [39,40,41]:

(1) $$E_{g}=1240/\lambda_{g},$$

(2) $$\chi (S)=\sqrt[{N}]{\chi_{1}^{n}\chi_{2}^{s}\ldots \chi_{n-1}^{p}\chi_{n}^{q}},$$

(3) $$E_{CB}=\chi (S)-E^{e}-\frac{1}{2}E_{g},$$

(4) $$E_{VB}=E_{CB}+E_{g},$$

where *E_g_*, *λ_g_*, *E^e^*, *E_CB_*, and *E_VB_* represent the bandgap, threshold wavelength, energy of free electrons on the hydrogen scale (~4.5 eV), and the *CB* and *VB* position, respectively. The values *χ*, *n*, and *N* represent the electronegativity of the constituent atom, number of species, and total number of atoms in the compound, respectively. The calculated *E_g_* of Co-TDC and CF are 1.72 eV and 1.57 eV, indicating that the CF hybrid possesses a narrower bandgap, and thus requires less excitation energy. Thereby, Figure 4b depicts the photocatalytic mechanism of CF under visible light illumination. The photo-generated electrons in CB cannot reduce O_2_ to ·O_2_^−^ because the *E_CB_* of Co_3_O_4_ and Fe_2_O_3_ are more positive than *E*(O_2_/·O_2_^−^) (−0.33 V vs. NHE), while the photo-generated holes are capable of oxidizing OH^−^ to hydroxyl radicals (·OH) as the *E_VB_* of Co_3_O_4_ and Fe_2_O_3_ are more negative than *E*(·OH/OH^−^) (1.97 V vs. NHE) [42,43,44]. In order to further verify this perception, quenching experiments were carried out using tert-butyl alcohol (TBA), ammonium oxalate (AO) and L-ascorbic acid (L-AA) to quench the ·OH, photo-generated holes and ·O_2_^−^, respectively [45,46]. It can be observed from Figure S6 that the degradation efficiency of RhB clearly decreases in presence of TBA and AO. Therefore, it can be deduced that the main reactive species involved in the photocatalytic reaction are photo-generated holes and hydroxyl radicals (·OH), which consequently degrade RhB molecules to colorless small molecules.

**Figure 5:**
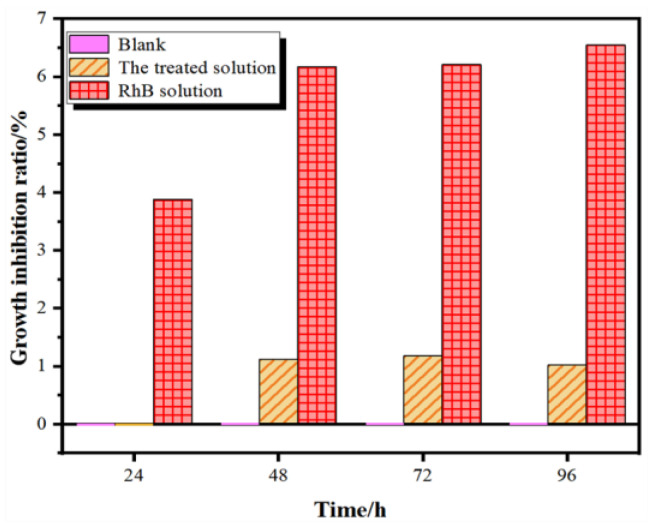
Time-dependent growth inhibition rates of *C. vulgaris* in different solutions.

In order to evaluate the ecological toxicity of the RhB solution before and after treatment, *Chlorella vulgaris* (FACHB-8) was used as the model aquatic organism being tested, and the toxicity of the residual RhB after the photocatalytic reaction was assessed according to its growth inhibition rate to *C. vulgaris*. A detailed experimental method for algae density measurement is presented in the Supplementary Materials, which could be referred to as the standard GBT 21805-2008 [47]. As exhibited in Figure 5, the growth of *C. vulgaris* was significantly suppressed in the original RhB solution, and the inhibition rate doubled as time increases. In contrast, *C. vulgaris* could grow normally in the solution after reaction, and the remaining intermediate and final products showed a neglectable influence within 24 h. Even when the incubation time was extended to 96 h, the growth inhibition rate was still about 1%, which is only 15.6% of the original RhB solution. This demonstrates that the CF catalyst can effectively degrade and mineralize RhB molecules to nearly non-toxic products.

## 4. Conclusions

In summary, a highly active and stable Co_3_O_4_/Fe_2_O_3_ heterostructural photocatalyst was prepared by a facile MOF-templated method, with its structure, morphology and optical properties verified by XRD, XPS, SEM and UV-visible DRS methodology. The results indicate that the CF catalyst showed a strong visible-light absorption and high photocatalytic activity towards RhB degradation. By calculating the *CB* and *VB* position, it could be inferred that hydroxyl radicals and photo-generated holes were the dominant active species in the reaction. Furthermore, the 96 h growth inhibition rate of *C. vulgaris* by the treated RhB solution was 84.4% lower than the original solution, confirming the potential of the CF photocatalyst for the sunlight-driven long-term degradation of dye molecules into non-toxic and colorless ones.

## Data Availability

The data presented in this study are available on request from the corresponding author.

## Supplementary Materials

The followings are available online at https://www.mdpi.com/article/10.3390/nano12060904/s1, Figure S1: Fe 2p XPS spectrum of as-synthesized CF catalyst, Figure S2: SEM images of pure Co-TDC at different magnifications, Figure S3: SEM images of pure Fe_2_O_3_ at different magnifications, Figure S4: Elemental mapping profiles of as-synthesized CF catalyst: (a) O Kα1, (b) Co Kα1, (c) Fe Kα1, Figure S5: PL spectra of CF catalyst comparing with pure Fe_2_O_3_ at excitation wavelength of 355 nm, Figure S6: Photocatalytic degradation efficiency of RhB with and without quenching agent, Figure S7: Photocatalytic degradation efficiency of RhB by as-synthesized CF catalyst comparing with commercial Degussa/Evonik P25-TiO_2_ catalyst, Figure S8: Photocatalytic degradation efficiency of RhB by CF over a prolonged period of time; Experimental biota for toxicity test and algae density measurement.

## References

[1] Han J., Yang D., Hall D.R., Liu J., Sun J., Gu W., Tang S., Alharbi H.A., Jones P.D., Krause H.M. (2020). Toxicokinetics of Brominated Azo Dyes in the Early Life Stages of Zebrafish (*Danio rerio*) Is Prone to Aromatic Substituent Changes. Environ. Sci. Technol..

[2] Chen M., Jia Y., Li H., Wu Z., Huang T., Zhang H. (2021). Enhanced pyrocatalysis of the pyroelectric BiFeO_3_/g-C_3_N_4_ heterostructure for dye decomposition driven by cold-hot temperature alternation. J. Adv. Ceram..

[3] Cao Q., Liu X., Yuan K., Yu J., Liu Q., Delaunay J.-J., Che R. (2017). Gold nanoparticles decorated Ag(Cl,Br) micro-necklaces for efficient and stable SERS detection and visible-light photocatalytic degradation of Sudan, I. Appl. Catal. B Environ..

[4] Cheng Y.-F., Cao Q., Zhang J., Wu T., Che R. (2017). Efficient photodegradation of dye pollutants using a novel plasmonic AgCl microrods array and photo-optimized surface-enhanced Raman scattering. Appl. Catal. B Environ..

[5] Cao Q., Che R., Chen N. (2015). Scalable synthesis of Cu_2_S double-superlattice nanoparticle systems with enhanced UV/visible-light-driven photocatalytic activity. Appl. Catal. B Environ..

[6] Cao Q., Che R., Chen N. (2014). Facile and rapid growth of Ag_2_S microrod arrays as efficient substrates for both SERS detection and photocatalytic degradation of organic dyes. Chem. Commun..

[7] Lee K.M., Lai C.W., Ngai K.S., Juan J.C. (2016). Recent developments of zinc oxide based photocatalyst in water treatment technology: A review. Water Res..

[8] Sadeghzadeh-Attar A. (2020). Photocatalytic degradation evaluation of N–Fe codoped aligned TiO_2_ nanorods based on the effect of annealing temperature. J. Adv. Ceram..

[9] Cao Q., Yuan K., Liu Q., Liang C., Wang X., Cheng Y.-F., Li Q., Wang M., Che R. (2015). Porous Au–Ag Alloy Particles Inlaid AgCl Membranes As Versatile Plasmonic Catalytic Interfaces with Simultaneous, in Situ SERS Monitoring. ACS Appl. Mater. Interfaces.

[10] Cao Q., Che R. (2014). Tailoring Au–Ag–S composite microstructures in one-pot for both SERS detection and photocatalytic degradation of plasticizers DEHA and DEHP. ACS Appl. Mater. Interfaces.

[11] Cheng J., Liu K., Li X., Huang L., Liang J., Zheng G., Shan G. (2020). Nickel-metal-organic framework nanobelt based composite membranes for efficient Sr^2+^ removal from aqueous solution. Environ. Sci. Ecotechnol..

[12] Cheng J., Liang J., Dong L., Chai J., Zhao N., Ullah S., Wang H., Zhang D., Imtiaz S., Shan G. (2018). Self-assembly of 2D-metal–organic framework/graphene oxide membranes as highly efficient adsorbents for the removal of Cs^+^ from aqueous solutions. RSC Adv..

[13] Dong J., Zhang X., Dong X., Ng K.H., Xie Z., Chen I.-W.P., Ng Y.H., Huang J., Lai Y. (2021). Coupled porosity and heterojunction engineering: MOF-derived porous Co_3_O_4_ embedded on TiO_2_ nanotube arrays for water remediation. Chemosphere.

[14] Cao Q., Hao S., Wu Y., Pei K., You W., Che R. (2021). Interfacial charge redistribution in interconnected network of Ni_2_P–Co_2_P boosting electrocatalytic hydrogen evolution in both acidic and alkaline conditions. Chem. Eng. J..

[15] Li R., Fu Q., Zou X., Zheng Z., Luo W., Yan L. (2020). Mn–Co–Ni–O thin films prepared by sputtering with alloy target. J. Adv. Ceram..

[16] Zhang L., Liu Y., Tan T.T., Liu Y., Zheng J., Yang Y., Hou X., Feng L., Suo G., Ye X. (2020). Thermoelectric performance enhancement by manipulation of Sr/Ti doping in two sublayers of Ca_3_Co_4_O_9_. J. Adv. Ceram..

[17] Li H., Zhang H., Thayil S., Chang A., Sang X., Ma X. (2021). Enhanced aging and thermal shock performance of Mn_1.95−*x*_Co_0.21_Ni_0.84_Sr*_x_*O_4_ NTC ceramics. J. Adv. Ceram..

[18] Liu L., Huang X., Wei Z., Duan X., Zhong B., Xia L., Zhang T., Wang H., Jia D., Zhou Y. (2021). Solvents adjusted pure phase CoCO_3_ as anodes for high cycle stability. J. Adv. Ceram..

[19] Song B., Yuan K., Wei Y., Chen D., Meng F., Cao Q., Song M., Liu H. (2021). In-furnace control of arsenic vapor emissions using Fe_2_O_3_ microspheres with good sintering resistance. Environ. Sci. Technol..

[20] Ye F., Dai H., Peng K., Li T., Chen J., Chen Z., Li N. (2020). Effect of Mn doping on the microstructure and magnetic properties of CuFeO_2_ ceramics. J. Adv. Ceram..

[21] Phor L., Chahal S., Kumar V. (2020). Zn^2+^ substituted superparamagnetic MgFe_2_O_4_ spinel-ferrites: Investigations on structural and spin-interactions. J. Adv. Ceram..

[22] Chen C., Wang Y., Li Z., Liu C., Gong W., Tan Q., Han B., Yao F., Wang K. (2021). Evolution of electromechanical properties in Fe-doped (Pb,Sr)(Zr,Ti)O_3_ piezoceramics. J. Adv. Ceram..

[23] Li J., Tang X., Liu Q., Jiang Y., Tang Z. (2021). Resistive switching and optical properties of strontium ferrate titanate thin film prepared *via* chemical solution deposition. J. Adv. Ceram..

[24] Hao S., Liu J., Cao Q., Zhao Y., Zhao X., Pei K., Zhang J., Chen G., Che R. (2020). In-situ electrochemical pretreatment of hierarchical Ni_3_S_2_-based electrocatalyst towards promoted hydrogen evolution reaction with low overpotential. J. Colloid Interface Sci..

[25] Hao S., Cao Q., Yang L., Che R. (2020). Morphology-optimized interconnected Ni_3_S_2_ nanosheets coupled with Ni(OH)_2_ nanoparticles for enhanced hydrogen evolution reaction. J. Alloys Compd..

[26] Cao Q., Yu J., Yuan K., Zhong M., Delaunay J.-J. (2017). Facile and Large-Area Preparation of Porous Ag_3_PO_4_ Photoanodes for Enhanced Photoelectrochemical Water Oxidation. ACS Appl. Mater. Interfaces.

[27] Yuan K., Wang C.-Y., Zhu L.-Y., Cao Q., Yang J.-H., Li X.-X., Huang W., Wang Y.-Y., Lu H.-L., Zhang D.W. (2020). Fabrication of a Micro-Electromechanical System-Based Acetone Gas Sensor Using CeO_2_ Nanodot-Decorated WO_3_ Nanowires. ACS Appl. Mater. Interfaces.

[28] Yuan K.-P., Zhu L.-Y., Cao Q., Ma H.-P., Tao J.-J., Huang W., Lu H.-L. (2020). ALD-based hydrothermal facile synthesis of a dense WO_3_@TiO_2_–Fe_2_O_3_ nanodendrite array with enhanced photoelectrochemical properties. J. Mater. Chem. C.

[29] Yuan K., Cao Q., Lu H.-L., Zhong M., Zheng X., Chen H.-Y., Wang T., Delaunay J.-J., Luo W., Zhang L. (2017). Oxygen-deficient WO_3–*x*_@TiO_2–*x*_ core-shell nanosheets for efficient photoelectrochemical oxidation of neutral water solutions. J. Mater. Chem. A.

[30] Yuan K., Cao Q., Li X., Chen H.-Y., Deng Y., Wang Y.-Y., Luo W., Lu H.-L., Zhang D.W. (2017). Synthesis of WO_3_@ZnWO_4_@ZnO–ZnO hierarchical nanocactus arrays for efficient photoelectrochemical water splitting. Nano Energy.

[31] Lassoued A., Lassoued M.S., Dkhil B., Ammar S., Gadri A. (2018). Synthesis, photoluminescence and magnetic properties of iron oxide (*α*-Fe_2_O_3_) nanoparticles through precipitation or hydrothermal methods. Phys. E Low Dimens. Syst. Nanostructures.

[32] Zhang D., Liu T., Cheng J., Cao Q., Zheng G., Liang S., Wang H., Cao M.S. (2019). Lightweight and high-performance microwave absorber based on 2D WS_2_–RGO heterostructures. Nano Micro Lett..

[33] Cao Q., Yu J., Cao Y., Delaunay J.-J., Che R. (2021). Unusual effects of vacuum annealing on large-area Ag_3_PO_4_ microcrystalline film photoanode boosting cocatalyst- and scavenger-free water splitting. J. Mater..

[34] Cao Q., Cheng Y.-F., Bi H., Zhao X., Yuan K., Liu Q., Li Q., Wang M., Che R. (2015). Crystal defect-mediated band-gap engineering: A new strategy for tuning the optical properties of Ag_2_Se quantum dots toward enhanced hydrogen evolution performance. J. Mater. Chem. A.

[35] Lima N.A., Alencar L.D.S., Siu-Li M., Feitosa C.A.C., Mesquita A., M’peko J.-C., Bernardi M.I.B. (2020). NiWO_4_ powders prepared *via* polymeric precursor method for application as ceramic luminescent pigments. J. Adv. Ceram..

[36] Luchechko A., Shpotyuk Y., Kravets O., Zaremba O., Szmuc K., Cebulski J., Ingram A., Golovchak R., Shpotyuk O. (2020). Microstructure and luminescent properties of Eu^3+^-activated MgGa_2_O_4_:Mn^2+^ ceramic phosphors. J. Adv. Ceram..

[37] Liu N., Mei L., Bin J., Zhang Z., Peng Z. (2021). Effect of anionic group [SiO_4_]^4−^/[PO_4_]^3−^ on the luminescence properties of Dy^3+^-doped tungstate structural compounds. J. Adv. Ceram..

[38] Cao Q., Che R. (2014). Synthesis of near-infrared fluorescent, elongated ring-like Ag_2_Se colloidal nanoassemblies. RSC Adv..

[39] Li C., Cao Q., Wang F., Xiao Y., Li Y., Delaunay J.-J., Zhu H. (2018). Engineering graphene and TMDs based van der Waals heterostructures for photovoltaic and photoelectrochemical solar energy conversion. Chem. Soc. Rev..

[40] Shao Y., Feng K., Guo J., Zhang R., He S., Wei X., Lin Y., Ye Z., Chen K. (2021). Electronic structure and enhanced photoelectrocatalytic performance of Ru*_x_*Zn_1−*x*_O/Ti electrodes. J. Adv. Ceram..

[41] Zhong M., Feng Q., Yuan C., Liu X., Zhu B., Meng L., Zhou C., Xu J., Wang J., Rao G. (2021). Photocurrent density and electrical properties of Bi_0.5_Na_0.5_TiO_3_–BaNi_0.5_Nb_0.5_O_3_ ceramics. J. Adv. Ceram..

[42] Siahroudi M.G., Daryakenari A.A., Molamahaleh Y.B., Cao Q., Daryakenari M.A., Delaunay J.-J., Siavoshi H., Molaei F. (2020). Ethylene glycol assisted solvo-hydrothermal synthesis of NGr–Co_3_O_4_ nanostructures for ethanol electrooxidation. Int. J. Hydrogen Energy.

[43] Yu J., Wang J., Long X., Chen L., Cao Q., Wang J., Qiu C., Lim J., Yang S. (2021). Formation of FeOOH Nanosheets Induces Substitutional Doping of CeO_2−*x*_ with High-Valence Ni for Efficient Water Oxidation. Adv. Energy Mater..

[44] Li Z., Dong T., Zhang Y., Wu L., Li J., Wang X., Fu X. (2007). Studies on In(OH)*_y_*S*_z_* solid solutions: Syntheses, characterizations, electronic structure, and visible-light-driven photocatalytic activities. J. Phys. Chem. C.

[45] Zhao Y., Song M., Cao Q., Sun P., Chen Y., Meng F. (2020). The superoxide radicals’ production *via* persulfate activated with CuFe_2_O_4_@Biochar composites to promote the redox pairs cycling for efficient degradation of *o*-nitrochlorobenzene in soil. J. Hazard. Mater..

[46] Meng F., Song M., Song B., Wei Y., Cao Q., Cao Y. (2020). Enhanced degradation of Rhodamine B *via α*-Fe_2_O_3_ microspheres induced persulfate to generate reactive oxidizing species. Chemosphere.

[47] Pei Z.-T., Xu R.-R., Liu H.-Y., Wang W.-Q., Zhang M., Zhang L.-L., Zhang J., Wang W.-Q., Yu R., Sun L.-W. (2020). Development and application of a novel whole sediment toxicity test using immobilized sediment and *Chlorella vulgaris*. Ecotoxicol. Environ. Saf..

