# Supplementary material for: Metal–Organic-Framework-Derived Ball-Flower-like Porous Co3O4/Fe2O3 Heterostructure with Enhanced Visible-Light-Driven Photocatalytic Activity
Source: Nanomaterials (Basel). 2022 Mar 9;12(6):904. doi: 10.3390/nano12060904 (PMC8951189; doi:10.3390/nano12060904)
Supplement: Supplementary file 1 [file nanomaterials-12-00904-s001.zip › nanomaterials-1616517-supplementary.pdf]

nanomaterials

Supplementary Materials

Metal–Organic-Framework-Derived Ball-Flower-like Porous Co_3O_4/Fe_2O_3 Heterostructure with Enhanced Visible-Light-Driven Photocatalytic Activity

Qi Cao [1,†,*], Qingqing Li [2,†], Zhichao Pi [3], Jing Zhang [1], Li-Wei Sun [1], Junzhou Xu [1], Yunyi Cao [4], Junye Cheng [5,*] and Ye Bian [1,*]

[1] Key Laboratory of Energy Thermal Conversion and Control of Ministry of Education, School of Energy and Environment, Wuxi Engineering Research Center of Taihu Lake Water Environment, Southeast University, Nanjing 210096, China; 213151155@seu.edu.cn (J.Z.); liwei-sun@seu.edu.cn (L.-W.S.); 213193744@seu.edu.cn (J.X.)

[2] Department of Chemistry, College of Sciences, Nanjing Agricultural University, Nanjing 210095, China; 2019203072@njau.edu.cn

[3] State-Operated Wuhu Machinery Plant, Wuhu 241099, China; pzc0279@163.com

[4] Department of Intelligent Development Platform, Laundry Appliances Business Division of Midea Group, Wuxi 214028, China; caoyy12@midea.com

[5] School of Science and Engineering, The Chinese University of Hong Kong, Shenzhen 518172, China

* Correspondence: qicao@seu.edu.cn (Q.C.); junye.cheng@polyu.edu.hk (J.C.); yebian@seu.edu.cn (Y.B.)

† These authors contributed equally to this work.

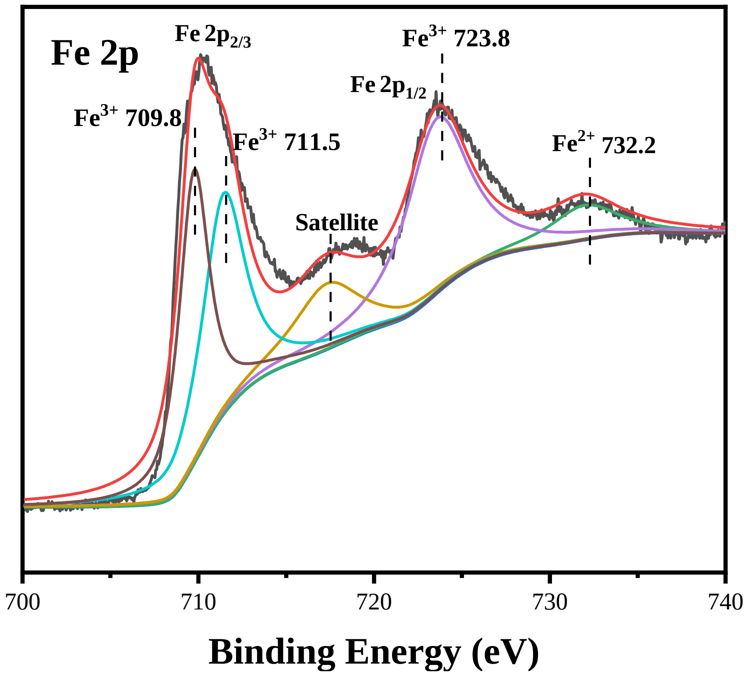

Figure S1. Fe 2p XPS spectrum of as-synthesized CF catalyst.

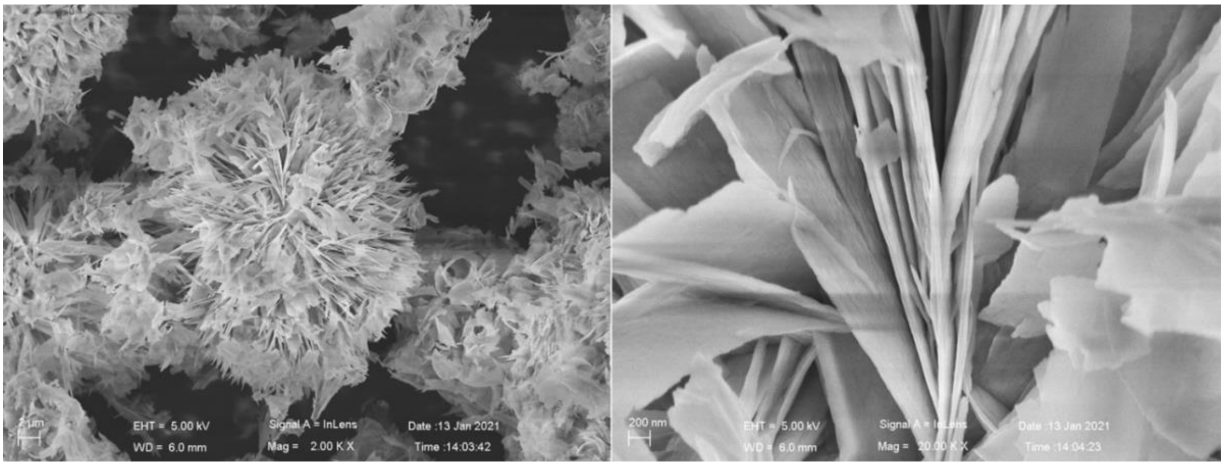

Figure S2. SEM images of pure Co-TDC at different magnifications.

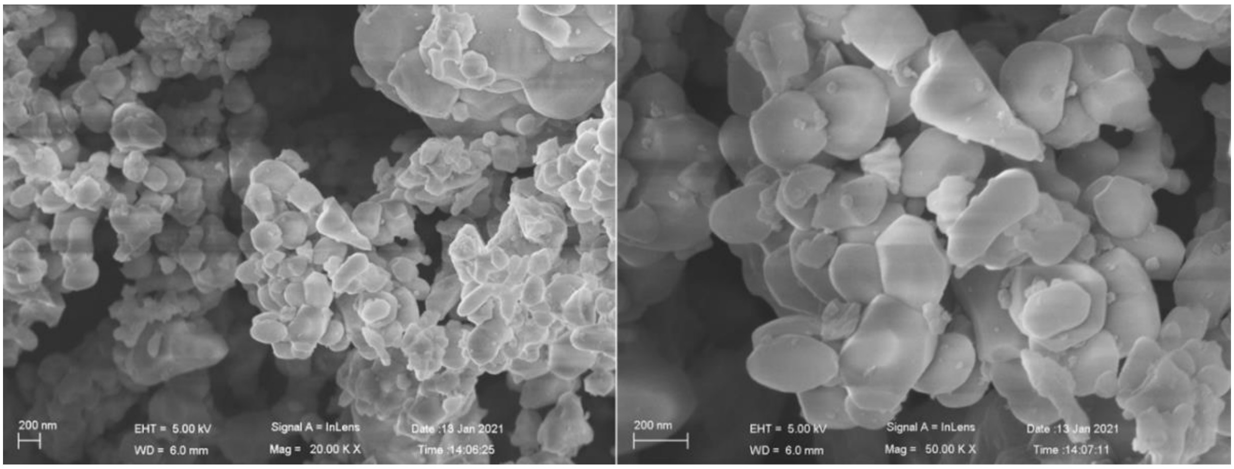

Figure S3. SEM images of pure Fe_2O_3 at different magnifications.

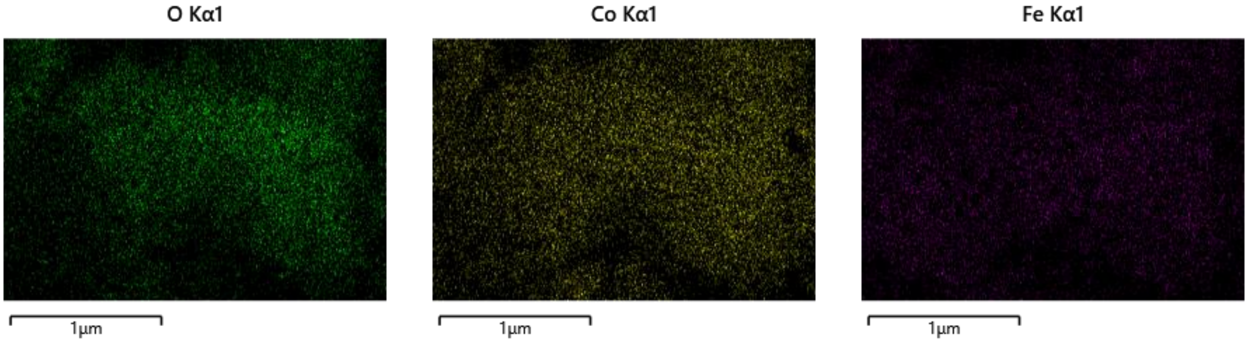

(**a**) (**b**) (**c**)

Figure S4. Elemental mapping profiles of as-synthesized CF catalyst: (**a**) O Kα1, (**b**) Co Kα1, (**c**) Fe Kα1.

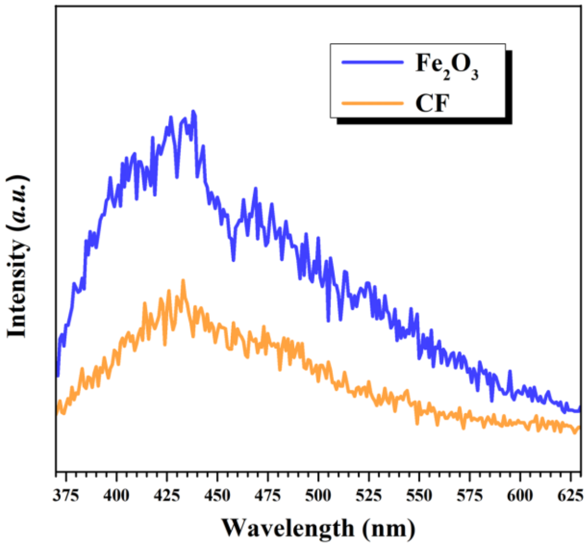

Figure S5. PL spectra of CF catalyst comparing with pure Fe_2O_3 at excitation wavelength of 355 nm.

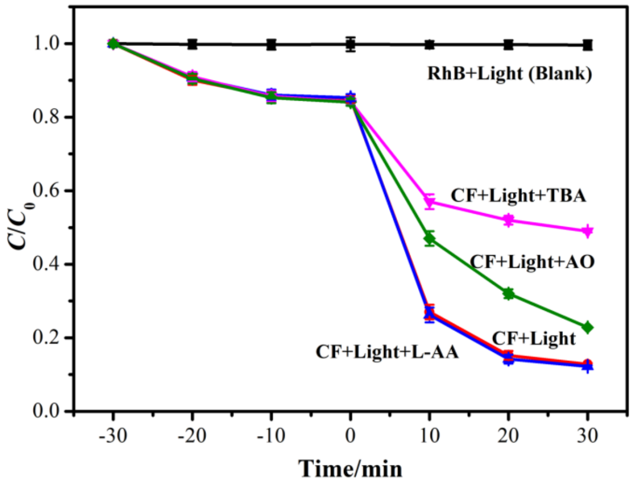

Figure S6. Photocatalytic degradation efficiency of RhB with and without quenching agent.

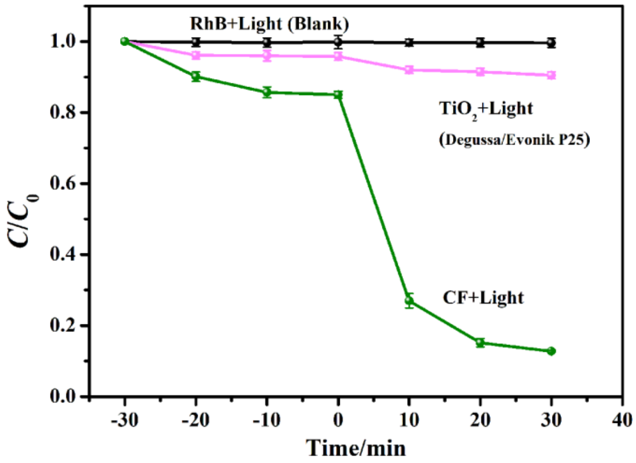

Figure S7. Photocatalytic degradation efficiency of RhB by as-synthesized CF catalyst comparing with commercial Degussa/Evonik P25-TiO_2 catalyst.

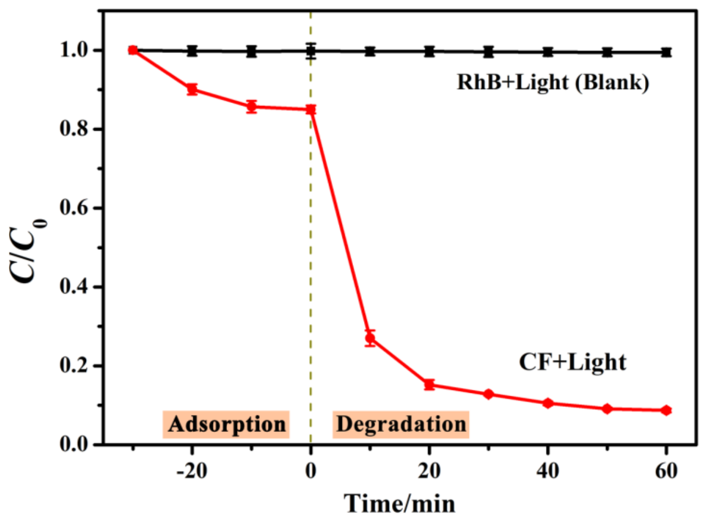

Figure S8. Photocatalytic degradation efficiency of RhB by CF over a prolonged period of time.

Experimental biota for toxicity test and algae density measurement

Chlorella vulgaris (FACHB-8) was obtained from the Freshwater Algae Culture Collection of the Institute of Hydrobiology at the Chinese Academy of Sciences (Wuhan, China). The blue-green algae medium (BG11) was used for culturing of *C. vulgaris*, and all the experiments were carried out at the same conditions: 40–80 μE m^{-2} s^{-1}, 25 ± 2 °C, pH = 7.1. Algae in the logarithmic growth phase were used for toxicity experiments. The algae density-absorbance curve was plotted based on the algae absorbance and the *C. vulgaris* counting results. A series density of the algae solutions was prepared, the absorbance was measured at 680 nm with a 7600 UV–visible spectrophotometer, and then the corresponding densities of *C. vulgaris* were counted under an Olympus optical microscope (CKX41, Japan) on a hemocytometer (Marienfeld, Germany). All samples were counted more than three times when the statistical results difference between each sample was less than 10%. In the subsequent experiments, the algae density could be calculated by measuring the absorbance based on the recorded absorbance-density curve. Except for the algae cell density in algae beads, it was counted under the microscope after beads were dissolved.
